# Subjectively salient faces differ from emotional faces: ERP evidence

**DOI:** 10.1038/s41598-024-54215-5

**Published:** 2024-02-13

**Authors:** Anna Żochowska, Anna Nowicka

**Affiliations:** Laboratory of Language Neurobiology, Nencki Institute of Experimental Biology, Polish Academy of Sciences, 3 Pasteur Street, 02-093 Warsaw, Poland

**Keywords:** Self, Saliency, Emotion, Familiarity, ERP, Psychology, Attention, Perception

## Abstract

The self-face is processed differently than emotional faces. A question arises whether other highly familiar and subjectively significant non-self faces (e.g. partner’s face) are also differentiated from emotional faces. The aim of this event-related potential (ERP) study was to investigate the neural correlates of personally-relevant faces (the self and a close-other’s) as well as emotionally positive (happy) and neutral faces. Participants were tasked with the simple detection of faces. Amplitudes of N170 were more negative in the right than in the left hemisphere and were not modulated by type of face. A similar pattern of N2 and P3 results for the self-face and close-other’s face was observed: they were associated with decreased N2 and increased P3 relative to happy and neutral faces. However, the self-face was preferentially processed also when compared to a close-other’s face as revealed by lower N2 and higher P3 amplitudes. Nonparametric cluster-based permutation tests showed an analogous pattern of results: significant clusters for the self-face compared with all other faces (close-other’s, happy, neutral) and for close-other’s face compared to happy and neutral faces. In summary, the self-face prioritization was observed, as indicated by significant differences between one’s own face and all other faces. Crucially, both types of personally-relevant faces differed from happy faces. These findings point to the pivotal role of subjective evaluation of the saliency factor.

## Introduction

Although the self is challenging to operationalize in cognitive and social neuroscience, it can be explored through investigations of self-referential processing^1^. In contrast to other types of self-related information, such as one’s own name or date of birth, the self-face is not shared with other people, and it is strongly tied to the sense of self-awareness^2,3^. This uniqueness makes the self-face a distinctive component of our physical identity^4,5^ and self-recognition may be viewed as the central process that enables maintaining the coherence of the self^6^. As humans are the subject of their own cognition, they are in the unique position of possessing years of detailed motor and sensory-feedback experiences about themselves which result in a highly-elaborated (not only visual but also multimodal) representation of the self-face^7^. Self-face recognition has been shown to be impaired in a variety of neurological or developmental disorders such as autism^8–10^ and schizophrenia^11–13^. In children suffering from autistic-spectrum disorders, mirror self-recognition is developmentally delayed and, in some cases, even absent^14^. Individuals with schizophrenia frequently perceive their own reflections in mirrors as independently alive, alien, or sinister^15^. Occasionally, they report even seeing nothing in the mirrored reflection^16^. Individuals scoring high on measures of schizotypal personality exhibit impairments in recognizing their own faces^17^.

Neural underpinnings of the self-face processing have been extensively investigated. Functional Magnetic Resonance Imaging (fMRI) studies showed that the self-face processing is mediated by cortical midline structures^18,19^ and increased activation of the medial prefrontal cortex and anterior cingulate cortex was typically reported^3,20,21^. Event-related potential (ERP) studies, in turn, provided valuable information about time-course of brain activity associated with the processing of one’s own face. Differences between one’s own face and other faces was found in amplitudes of early and late ERP components: N170, N2, P3, and LPP (see below).

The occipito-temporal N170 is typically linked to stimulus-category discrimination and it is enhanced for faces compared to other non-face objects^22,23^. Thus it is asserted to be face-specific^24–26^ and there is widespread agreement that N170 represents the analysis of structural information of faces^27–30^. Numerous findings reported that the N170 is relatively unaffected by face familiarity, with similar N170 potentials elicited by both familiar and unfamiliar faces^31,32^, regardless of their relevance to the task^27^. A recent review^33^ highlighted the fact that this familiarity effect was found only in about half of the analyzed N170 studies. Although some studies have presented evidence that this component is larger (i.e. more negative) to the self-face when compared to other faces, whether familiar or not^34–39^, reports of no self-face effect on the N170 component were even more frequent^9,40–50^.

On the other hand, some studies showed that initial indications of self-face discrimination, distinguishing between one’s own face and others, occur outside visual areas. This is manifested by a reduction in midfrontal N2 for one’s own face^39,40,49,51–53^ or as an increase of P3 in central-parietal areas^9,10,41,45,49,50,53–56^. An increase of the midfrontal N2 often signifies a more pronounced involvement of certain forms of executive control^57^. Therefore, a smaller stimulus-related amplitude increase of the midfrontal N2 under self-related conditions, compared to the self-unrelated conditions, may suggest a reduced engagement of executive control in visual encoding and response execution^57–59^.

The P3, on the other hand, has been associated with various cognitive functions, including context updating, allocation of attentional resources, and associative memory processes (for review see^60^). Crucially, the P3 component reflects the cognitive evaluation of stimulus significance, a process that can be triggered by both active and passive attention^61–64^. Numerous ERP studies employing different experimental paradigms consistently indicate that the P3 is influenced by the familiarity of faces, with the processing of one’s own face resulting in a significantly higher P3 amplitude than processing of other faces^10,27,32,40–42,50,53–56,65–67^.

Only a few studies reported an increased Late Positive Potential (LPP) in response to the self-face compared to other faces, regardless of their familiarity^50,54,55^. The LPP is commonly associated with a global, temporary enhancement of attention, facilitating the in-depth processing of salient stimuli^68,69^. The heightened LPP in response to the self-face may be attributed not only to its saliency^2,70–72^ but also to the process of self-reflection, as viewing one’s own face tends to evoke a particularly unique emotional response^20^.

Hence, results of previous studies clearly indicate that the familiarity of processed faces matters as differences in early and late ERPs components were noted between the self-face (representing an extremely familiar face) and faces that were less familiar or unfamiliar. Importantly, factors beyond familiarity can be identified as driving the preferential processing of any self-relevant information, such as emotional significance and subjective salience. Therefore, it can be argued that the emotional significance may also contribute to the aforementioned pattern of findings and may determine the prioritized self-face processing. Existing evidence suggests that all self-related information can be perceived to varying degrees as emotional^67^. It is conceivable that the personal relevance of a specific stimulus determines its subjective emotional or neutral evaluation. This proposition is supported by fMRI studies revealing increased activation in the medial prefrontal cortex and anterior cingulate cortex, associated with both general emotion processing^73,74^ and self-face processing^3,20,21^. This implies that exposure to the self-face may effectively induce introspection and emotional reactions.

Nevertheless, ERP studies that directly compared the self-face and emotional faces processing showed substantial differences between those two types of faces^54,75^. The earlier of those studies investigated the processing of the self-face, emotionally negative, and neutral unknown faces presented as deviant stimuli in the odd-ball procedure^75^. The results demonstrated significantly enhanced early (P2) and late (P3) ERP components in response to the self-face when compared to emotionally negative faces. Considering that individuals typically harbor a more positive than negative attitude toward themselves^76,77^ the comparison of the self-face to a happy face appears to be more ecologically valid. Therefore, in another study, besides emotionally negative (fearful) faces, emotionally positive (happy) faces were incorporated^54^. The findings from this study indicated significantly increased P3 amplitudes in response to the self-face when compared to both types of emotional as well as neutral faces.

The aforementioned findings could be attributed either to differences in the levels of familiarity between the self and emotional (unknown) faces or to the heightened psychological significance and subjective relevance of one’s own face^54^. Nonetheless, it raises the question of whether similar effects to those observed for the self-face versus emotional faces would emerge for personally relevant but non-self faces. These faces, akin to one’s own face, embody a blend of extreme familiarity and subjective significance/emotional load factors. Responses to the above question may unveil whether the distinctions between the self-face and emotional faces are exclusive to the self or whether other highly familiar and significant faces undergo similar processing to the self-face. Thus, they may contribute to the ongoing discussion regarding whether the self is a higher-order function or a fundamental function of the brain^78^, and may provide some additional arguments in favor of one of the contrasting viewpoints^79^.

Therefore, the present ERP study aimed at investigating the processing of subjectively significant, personally-relevant faces (self, close-other’s) in comparison to emotional unknown faces. Instead of a predefined individual (e.g., a mother, a friend), each participant freely selected their close-other, representing the most significant person in their life at the time of the experiment. This operationalization of a close-other has been utilized in numerous prior studies on the theme of self-prioritization^9,10,50,55,56,80–82^. Similarly to one’s own face, a close-other’s face is a highly important and salient visual stimulus encountered frequently in everyday life^82^.

The other category of faces contained images of happy faces, chosen as an appropriate control condition to the self-face, given the self-positivity bias^77,83–85^ and the theory of implicit positive association (IPA) with the self^76^. The self-positivity bias is notably robust and has been consistently observed across diverse populations, spanning differences in age, gender, psychopathology, and culture^86–88^. While the self-positivity bias pertains to various self-related domains, the IPA theory specifically targets self-face processing. Its fundamental premise is that an implicit positive association with the self underlies its advantage in face recognition. In other words, the process of recognizing one’s own face activates positive attributes in the self-concept, facilitating responses to the self-face and resulting in a self-advantage in face recognition. Neutral unknown faces, devoid of salience and self-relevance, were included as an additional control condition.

Therefore, the current study investigated the neural underpinnings of the processing of one’s own, a close-other’s, emotionally positive, and neutral faces using the ERP method. Participants were tasked with the simple detection of faces. Such a task was used in numerous previous studies on the face processing, yielding positive outcomes, i.e. detection of one’s own face resulted in heightened P3 amplitudes compared to all other faces, whether familiar or not^9,50,53,54,56^. Therefore, the self-preference effect was observable even in the absence of an explicit requirement for intentional discrimination among the presented stimuli.

The analysis of ERPs was focused on components commonly addressed in studies investigating the processing of self-face and emotional faces: N170^36,38,39,89^, N2^39,40,49,90^, and P3^48,50,67,91^. In addition to ERP analyses, spatio-temporal cluster-based permutation tests^92^ were employed to examine the distinctive patterns of activity evoked by different face types. This method is widely acknowledged for enabling unbiased comparisons of EEG signals recorded under different experimental conditions at all electrodes and time points. It effectively controls for multiple comparisons and maximizes power by utilizing the cluster structure of the data as its primary test statistic.

In sum, we expected that differences between the processing of subjectively significant and subjectively non-significant faces would be observed in amplitudes of both early and late ERP components of interest as well as in results of cluster-based permutation tests.

## Materials and methods

### Ethics statement

This study was conducted with the approval of the Human Ethics Committee of the Institute of Applied Psychology at Jagiellonian University (Cracow, Poland). The study was carried out in accordance with the guidelines and regulations of the Declaration of Helsinki. All participants provided written informed consent before to the study and received financial compensation for their participation.

### Participants

Thirty-seven participants (20 females, 17 males) between the ages of 21 and 34 (*M* = 28.3; *SD* = 3.2) took part in the study. Thirty-five participants were right-handed as verified by the Edinburgh Handedness Inventory^93^. Only participants with normal or corrected-to-normal vision using contacts and without distinctive facial marks were recruited. This criterion was implemented to ensure uniformity in visual stimuli standards, as each participant’s photograph was matched with images from the Chicago Face Database—CFD^94^. The database includes images of faces without glasses and without visible facial marks. All participants reported no history of mental or neurological diseases.

The required sample size was estimated using the G*Power 3 software^95^. Estimation was conducted for a repeated measures ANOVA with four levels (estimated effect size f = 0.25, α = 0.05, β = 0.90, and non-sphericity correction ε = 1.0), resulting in a sample size estimate of 30 participants. Considering the potential risk of data loss or exclusion, the group size was increased to 37.

### Stimuli

In the current study, the set of stimuli was individually tailored for each participant. It comprised single face images of four types: the self-face, a close-other’s face, an emotionally positive (happy/smiling) face, and a neutral face. The sex of other faces was matched to each participant’s sex in order to control the between-category variability in attentional effects. Participants freely selected the close-other based on their subjective high level of closeness and subjective significance to avoid the situation in which a pre-defined close-other might not truly be considered close by the participant. This approach has been employed in numerous previous studies^9,10,53,55,56,80,82^. The only restriction put on the selection of a close-other was that they be of the same sex and have no distinctive facial marks.

The face of each participant and their close-other was photographed before to the study. All participants and their close-others were invited to the lab to have a photograph of their face taken in a standardized environment (the same background and lightning conditions). Participants were asked to maintain a neutral facial expression during the photography session. Photographs of emotional and neutral faces were taken from the CFD database^94^. The sex of faces from the CFD database was matched to each subject’s sex in order to control for the between category variability. Different images of emotional and neutral faces were utilized in individual sets of stimuli to prevent the potential influence of a single selected image on the pattern of brain activity. In each stimuli set the CFD images represented two different identities. For example, if an image of a happy face of a specific actor was chosen, the images of neutral faces came from a different actor.

Images within each stimuli set (i.e. the self-face image, a close-other’s face image, and selected CDF images) were extracted from the background, grey-scaled, cropped to include only the facial features (i.e. the face oval without hair), resized to subtend 6.7° × 9.1° of visual angle, and equalized for mean luminance using Photoshop® CS5 (Adobe, San Jose, CA). Contrast and spatial frequencies were not normalized in the images, as these procedures can introduce substantial distortions. All faces were presented against a black background. None of the stimuli were shown to the participants before the experiment. The image of each participant’s face was removed from the procedure computer at the end of the experimental session.

### Procedure

Upon arrival, participants completed the Edinburgh Handedness Inventory (93 Oldfield, 1971). Subsequently, they were seated in a comfortable chair in a dimly lit and sound-attenuated room and were positioned 57 cm from the computer monitor (DELL Alienware AW2521HFL, Round Rock, Texas, USA). After the placement of the electrode cap (ActiCAP, Brain Products, Munich, Germany), participants used an adjustable chinrest to maintain a stable head position. Presentation software (Version 18.2, Neurobehavioral Systems, Albany, CA) was employed for stimuli presentation.

Participants engaged in a simple detection task: irrespective of the presented image (self-face, close-other’s, emotional, or neutral face), they were instructed to pressh the same response button (Cedrus response pad RB-830, San Pedro, USA) as quickly as possible. Upon reading the instructions displayed on the screen, participants initiated the experiment by pressing a response button.

**Figure 1:**
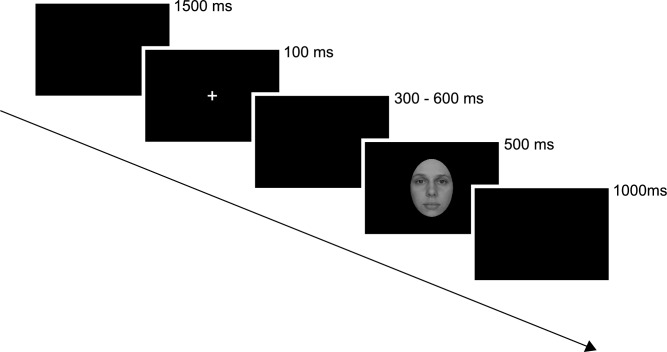
Schematic presentation of the experimental procedure. Four types of faces (self, close-other’s, happy, unknown) were intermixed and presented pseudo-randomly. Participants were tasked with simple detection. The example image is a photograph of one of the co-authors.

Each trial commenced with a blank screen, displayed for 1500 ms. Subsequently, a white cross (subtending 0.5° × 0.5° of visual angle) was centrally presented for 100 ms, then followed by a blank screen lasting either 300, 400, 500 or 600 ms at random. Following this, a stimulus was presented for 500 ms, succeeded by a blank screen for 1000 ms (see Fig. 1). The number of repetitions for each face category was 72. The order of stimuli presentation was pseudo-randomized, ensuring that no more than two stimuli of the same category were displayed consecutively. A break was scheduled in the middle of experiment to prevent participants’ fatigue. It lasted 1 min, unless the participant chose to commence the second part of the experiment earlier. On average, participants required 20 min to complete the entire experiment.

The experimental protocol was approved by the Human Ethics Committee of the Institute of Applied Psychology at Jagiellonian University (Cracow, Poland). The study was carried out in accordance with the guidelines and regulations of the Declaration of Helsinki, and written informed consent was obtained from each participant before the experiment.

### EEG recording

The EEG was continuously recorded with 62 Ag–AgCl electrically shielded electrodes mounted on an elastic cap (ActiCAP, Brain Products, Munich, Germany) and positioned according to the extended 10–20 system. Two additional electrodes were placed on the left and right earlobes. The data were amplified using a 64-channel amplifier (BrainAmp MR plus; Brain Products, Germany) and digitized at a 500-Hz sampling rate using BrainVision Recorder software (Brain Products, Munich, Germany). EEG electrode impedances were kept below 10 kΩ. The EEG signal was recorded against an average of all channels calculated by the amplifier hardware.

### Behavioral data analysis

Responses within a 100–1000 ms time-window after stimulus onset were judged as correct and analyzed using JASP software packages^96^. Because RTs exhibited right-skewed distributions, median RTs were analyzed. A mixed model ANOVA was performed with type of face (self, close-other’s, emotional, neutral) as a within-subject factor on the number of correct responses as well as median RTs. In the description of results, mean ± SD were provided for each experimental condition. One date set was excluded due to a very low number of correct responses (10%). Thus, analyses were run on 36 data sets.

### ERP analysis

The EEG data underwent offline analysis using BrainVision Analyzer® software (Version 2.2, Brain Products, Gilching, Germany). The EEG signals from 62 channels were re-referenced offline to the algebraic average recorded at the left and right earlobes. Subsequently, the data were notch-filtered at 50 Hz and band-pass-filtered from 0.01 to 30 Hz using a 2nd order Butterworth filter. After this re-referencing and filtering process, Independent Component Analysis—ICA^97^ was applied to correct ocular artifacts. Following the decomposition of each dataset into maximally statistically independent components, components representing eye blinks were excluded based on a visual inspection of the component’s topography^98^. Using the reduced component-mixing matrix, the remaining ICA components were multiplied and back-projected to the data, resulting in a set of EEG data free from ocular artifacts. Subsequently, the EEG signal was segmented into epochs of 1200 ms length, spanning from − 200 before to 1000 ms after stimulus onset. The subsequent step involved a semi-automatic artifact rejection procedure that discarded trials surpassing the following thresholds: the maximum permitted voltage step per sampling point was 50 µV, the maximum allowed absolute difference between two values in the segment was 200 µV, and the lowest permitted activity within a 100 ms interval was 0.5 µV. Finally, the epochs underwent baseline correction by subtracting the mean of the pre-stimulus period of 200 ms.

Two datasets had to be excluded from the sample: one because of a low number of trials remaining after artifact rejection (the exclusion threshold was set at less than 50% of trials), and the other due to technical malfunction. Consequently, all ERP analyses were conducted on the group of 35 participants (19 females, 16 males). The mean number of segments averaged for each type of face was as follows: self-face = 67.5 ± 7.2, close-other’s face = 67.4 ± 7.0, happy face = 66.6 ± 6.9, and neutral face = 64.4 ± 6.9.

**Figure 2:**
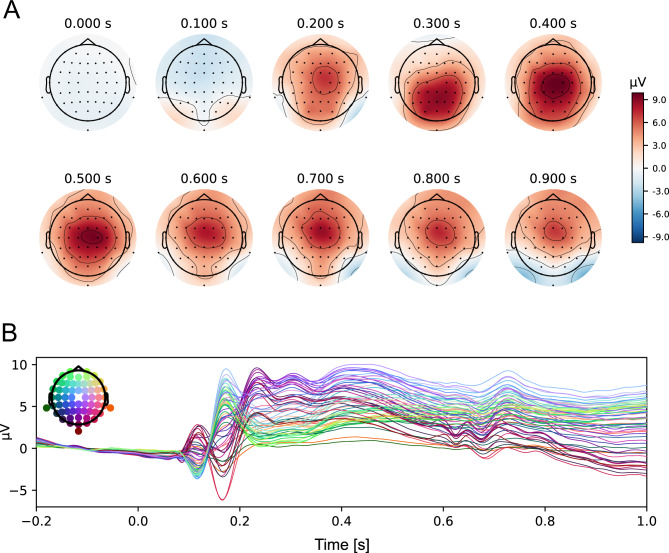
Maps of the topographical distribution of activity collapsed for all experimental conditions: self-, close-other’s, happy, and neutral face (**A**). Numbers above each map (i.e. 0.000 s, 0.100 s, etc.) indicates the beginning of the 100 ms time-interval for which the topography was depicted. Butterfly plot presenting grand-average ERPs for collapsed all experimental conditions, at all 62 active electrodes (**B**). The color of each ERP corresponds to the color of electrode site at which EEG signal was recorded and afterwards the ERP was calculated.

We utilized the topographical distribution of brain activity, averaged across all experimental conditions (all types of faces), to identify channels at which ERP components of interest reached maximum amplitude (see Fig. 2). This approach adhered to the general rule concerning electrode selection for ERP analyses, i.e., it must be orthogonal to potential differences between experimental conditions^99^. Based on the topographical maps as well as grand-averaged ERPs, collapsed for all conditions (self-face, close-other’s face, emotional face, neutral face), the following windows were chosen for the analysis of ERP components of interest: 100–200 ms for N170, 220–300 ms for N2, and 250–500 ms for P3 (Fig. 2). Electrodes within the region of maximal activity changes were selected: (1) N170—left cluster: PO7, P7, and right cluster: PO8, P8; (2) N2—midfrontal cluster: Fz, FCz; (3) P3—centro-parietal cluster: Pz, CPz, CP2, CP1. The data from these electrodes were pooled. This step is justified by the limited spatial resolution of EEG and high correlation between neighboring electrodes. Peak amplitudes were analyzed for N170, whereas for N2 and P3, mean amplitudes within the aforementioned time-windows were analyzed.

All statistical analyses were performed using the JASP software^96^. For N170 peak amplitudes, a mixed model ANOVA was performed with hemisphere (left, right) and type of face (self, close-other’s, emotional, neutral) as within-subject factors. For N2 and P3 mean amplitudes, repeated measures ANOVAs were performed with type of face (self, close-other’s, emotional, neutral) as within-subject. Adjustments for violations of sphericity were made for all effects with more than one degree of freedom in the numerator^100^. Post-hoc analyses were subjected to Bonferroni correction for multiple comparisons. In the description of results, mean ± SD (standard deviation) were provided for each experimental condition.

The traditional null-hypothesis significance-testing approach was complemented with Bayesian analysis methods and Bayes factors (BFs) were computed using JASP software^101^. It is worth noting that BF_10_ evaluates how strongly both alternative and null hypotheses are supported by the data. The medium prior scale (Cauchy scale 0.707) was used in all Bayesian tests. The interpretation of BFs followed the guidelines suggested by Lee and Wagenmakers^102^. To summarize, a BF_10_ between 1 and 3 implies anecdotal evidence in favor of H1, between 3 and 10 suggests moderate evidence, between 10 and 30 indicates strong evidence, between 30 and 100 suggests very strong, and values higher than 100 signify extreme evidence. As far as low values of BF_10_ are concerned, a BF_10_ between 0.33 and 1 indicates anecdotal evidence in favor of H0, between 0.1 and 0.33—moderate evidence, and between 0.03 and 0.1—strong evidence of the absence of an effect. Finally, a BF_10_ between 0.01 and 0.03 and lower than 0.01 indicates very strong and extreme evidence for the absence of an effect, respectively.

### Nonparametric cluster-based permutation tests

Cluster‐based permutation tests were chosen for their efficacy in addressing the multiple comparisons problem associated with analyses of high‐dimensional magnetoencephalographic and EEG data^103^. Unlike traditional ERP analyses, which typically focus on data recorded at a single electrode or a small set of pooled electrodes within a specific time window, cluster-based permutation tests allow for the comparison of different experimental conditions while considering all electrodes and time points^92^. Consequently, permutation tests offer a more comprehensive perspective on the commonalities and distinctions in the neural underpinnings of self-face and close-other’s faces versus other faces processing. The direct comparisons encompassed the following conditions: self vs. close-other’s, self vs. happy, self vs. neutral, close-other’s vs. happy, close-other’s vs. neutral, and happy vs. neutral. Employing clustering in both space and time, this analytical approach unveiled differences in the spatial distributions of activity over time among the tested conditions.

Results are presented with reference to an alpha level set at 0.05, and the cluster-based permutation tests were conducted using custom-made Python scripts.

## Results

### Behavioral results

The number of responses given within a 100–1000 ms time-window after stimulus onset was consistently high across all conditions: self-face—69.9 ± 5.9, close-other’s face—70.8 ± 5.3, happy face—70.4 ± 4.7, and neutral face—70.3 ± 5.3. The median RTs were as follows: the self-face—257.5 ± 29.4 ms, close-other’s face—258.5.8 ± 32.0, happy face—258.0 ± 33.5 ms, and neutral face—251.1 ± 31.7 ms. Overall, no significant effect were found in the RTs analysis (type of face: *F*_3,105_ = 0.231, *p* = 0.875, *η*_*p*_^2^ = 0.007). The lack of a significant effect of face type was further supported by BFs values indicating a moderate evidence in favor of H0 (self vs. close: BF_10_ = 0.213, self vs. happy: BF_10_ = 0.184, self vs. neutral: BF_10_ = 0.184, close vs. happy: BF_10_ = 0.184, close vs. neutral: BF_10_ = 0.251, happy vs. neutral: BF_10_ = 0.205).

### ERP results

#### N170

**Figure 3:**
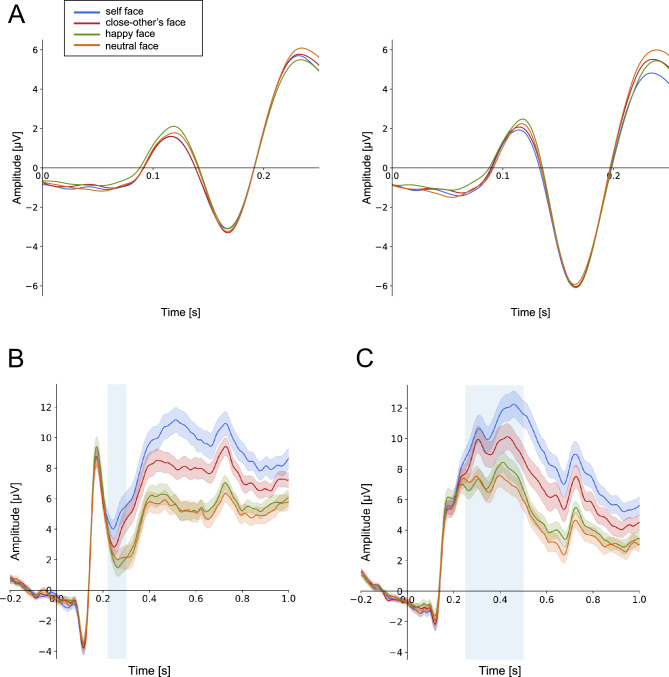
Grand average ERPs to self-, close-other’s, happy, and neutral faces. (**A**) N170 for pooled electrodes P7/PO7 (left side) and P8/PO8 (right side). Peak amplitude of this component was analyzed in the 100–200 ms time-window. (**B**) N2 for pooled electrodes Fz, FCz. (**C**) P3 for pooled electrodes Pz, CPz, CP2, and CP1. The analyzed time windows are marked by light-blue rectangles.

Figure 3 (panel A) displays the grand-average ERPs at pooled P7/PO7 and pooled P8/PO8. A mixed model ANOVA conducted on N170 amplitudes revealed a significant main effect of hemisphere, indicating that N170 amplitudes were more negative in the right hemisphere compared to the left hemisphere (− 6.6 ± 4.1 µV vs. − 4.2 ± 3.0 µV, *F*_1,34_ = 15.228, *p* < 0.001, *η*_*p*_^2^ = 0.309). However, all other effects were non-significant (type of face: *F*_3,102_ = 0.714, *p* = 0.546, *η*_*p*_^2^ = 0.021; type of face × hemisphere:* F*_3,102_ = 0.389, *p* < 0.761,* η*_*p*_^2^ = 0.011). Additionally, the non-significant effect of face type was further supported by BFs values, indicating moderate evidence in favor of the absence of an effect (self vs. close: BF_10_ = 0.212, self vs. happy: BF_10_ = 0.221, self vs. neutral: BF_10_ = 0.702, close vs. happy: BF_10_ = 0.132, close vs. neutral: BF_10_ = 0.207, happy vs. neutral: BF_10_ = 0.180).

#### N2

Grand-average ERPs (at pooled FCz and Fz) are presented in Fig. 3 (panel B). A repeated measures ANOVA conducted on N2 amplitudes revealed a significant main effect of the type of face (*F*_3,102_ = 16.837, *p* < 0.001,* η*_*p*_^2^ = 0.331). Post-hoc analyses demonstrated significant differences between N2 amplitudes for the self-face (5.1 ± 3.5 µV) compared to happy (2.5 ± 3.0 µV) and neutral (2.9 ± 3.1 µV) faces (*p* < 0.001, BF_10_ = 138,372.646 and *p* < 0.001, BF_10_ = 332.491, respectively). Similar effects were observed for a close-other’s face. N2 amplitudes to a close-other’s face (4.0 ± 3.3 µV) differed from those to a happy face (*p* = 0.002, BF_10_ = 22.287), with a statistical trend for a difference between close-other’s and neutral faces (*p* = 0.066, BF_10_ = 3.990). Furthermore, the self-face differed from a close-other’s face (*p* = 0.037, BF_10_ = 5.292), while the happy vs. neutral face comparison showed a non-significant difference (*p* > 0.9, BF_10_ = 0.496).

#### P3

Grand-average ERPs (at pooled CPz, CP1, CP2, and Pz) are presented in Fig. 3 (panel C). A repeated measures ANOVA conducted on P3 amplitudes showed a significant main effect of type of face (*F*_3,102_ = 45.520, *p* < 0.001,* η*_*p*_^2^ = 0.572). Post-hoc analyses indicated that P3 amplitude to the self-face (11.1 ± 4.7 µV) was significantly higher than P3 amplitudes to close-other’s (9.6 ± 4.7 µV), happy (7.7 ± 3.5 µV), and neutral (7.2 ± 3.8 µV) faces (all *ps* < 0.001; BF_10_ = 809.587, BF_10_ = 7,160,000, and BF_10_ = 9,820,000, respectively). P3 amplitude to the close-other’s face was higher than to happy and neutral faces (both *p*s < 0.001, BF_10_ = 151.451, and BF_10_ = 145,316.951, respectively). However, P3 amplitudes to happy and neutral faces did not differ (*p* > 0.9, BF_10_ = 0.474).

### Nonparametric cluster-based permutation tests

**Figure 4:**
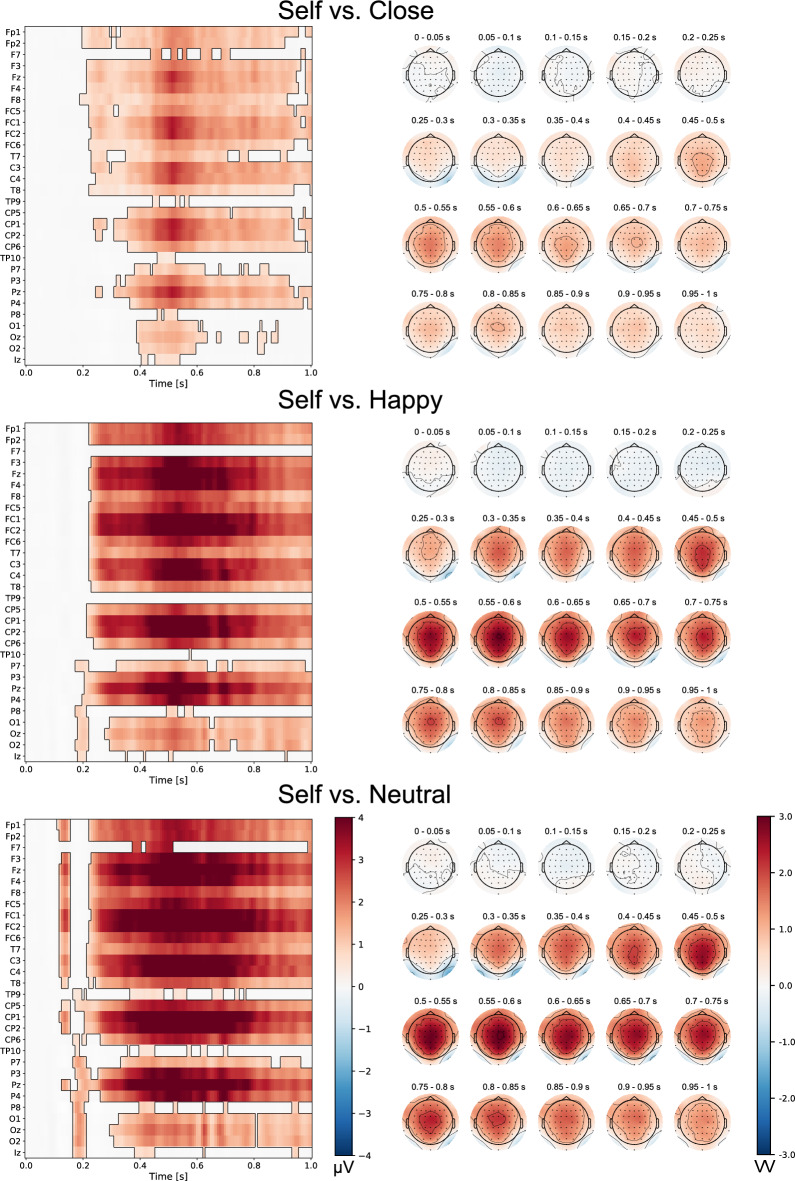
The results of cluster-based permutation tests for the self-face compared to other (close-other’s, happy, neutral) faces (left panel). Statistically significant differences between tested conditions are depicted (*p* < 0.05). The intensity of the color indicated the size of differences between the tested conditions. Results are shown for 30 (from 62) electrode sites (frontal, central, temporal, parietal, occipital, from the top to the bottom, respectively) and all time samples, i.e. from 0 (onset of the face image) till 1000 ms. Maps depicting the topographical distribution of difference waves are presented for consecutive 50 ms time-windows (right panel). Results for the other set of electrode sites are included in the Supplementary Information.

**Figure 5:**
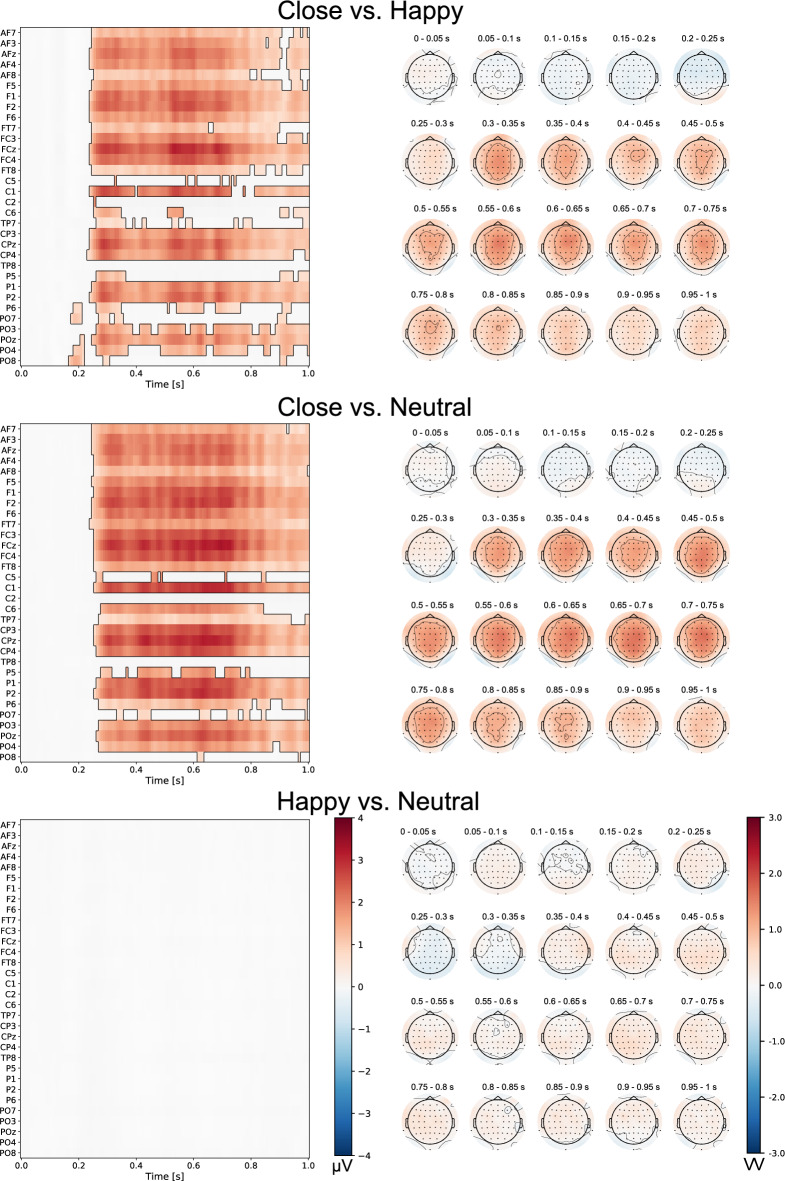
The results of cluster-based permutation tests for a close-other’s face compared to other (happy, neutral) faces and for happy vs. neutral face comparison (left panel). Statistically significant differences between tested conditions are depicted (*p* < 0.05). The intensity of the color indicated the size of differences between the tested conditions. Results are shown for 30 (from 62) electrode sites (frontal, central, temporal, parietal, occipital, from the top to the bottom, respectively) and all time samples, i.e. from 0 (onset of the face image) till 1000 ms (left panel). Maps depicting the topographical distribution of difference waves are presented for consecutive 50 ms time-windows (right panel). Results for other set of electrode sites are included in the Supplementary Information.

Nonparametric cluster‐based permutation analyses revealed a significant effect of the type of face (*p* < 0.05). Self-face processing exhibited notable differences from the processing of all other faces: close-other’s, happy, and neutral (Fig. 4). Similar pattern of findings were observed for a close-other’s face compared to happy and neutral faces (Fig. 5). In each instance, these significant clusters were widely distributed in space and time. Importantly, the time windows demonstrating substantial differences between the tested conditions aligned with those in which the N2 and P3 components were analyzed. Furthermore, differences between the self-face vs. other faces and a close-other’s face vs. other faces were observed at electrodes within the midfrontal and central-parietal regions—areas in which N2 and P3 amplitudes were analyzed. However, the comparison between happy face and neutral face revealed no significant cluster, also in line with ERP results (Fig. 5).

## Discussion

Human faces are generally ecologically salient stimuli^104^. However, they are not equally salient. As the self-face serves as the emblem of the self, its saliency surpasses that of other faces^4^. Neverthless, the subjective significance of a close-other’s face (e.g. partner’s or best friend’s face) is also very high and even comparable to that of one’s own face. The present study aimed to investigate the processing of these subjectively salient faces in comparison to emotional faces. The primary research question we sought to address was whether the effects observed for the self-face could also be identified for non-self faces that, akin to one’s own face, are subjectively significant and extremely familiar.

On the behavioral level, we observed no differences in RTs between experimental conditions. Notably, there was no RT effect for the self-face (i.e. no decreased RT to the self-face than to other faces) although it has been reported in previous studies (for a comprehensive review see^105^). It is crucial to emphasize that the task employed in our study was very simple (detection) and therefore did not necessitate overt discrimination of stimuli. Consequently, successful completion of the behavioral task did not demand in-depth processing of incoming information.

In some earlier studies involving such a task, the absence of RT differences between the self-face and other faces was also reported^9,50,53,54,56^. Moreover, in other tasks where the self-face was not in the focus of participants’ attention non-significant RT differences between faces were also observed^10,106^. However, it is essential to note that overt behavior reflects the final outcomes of a cascade of processes, beginning with sensory stimulation and culminating in a motor response. In contrast, the ERP method provides insights into the neural mechanisms that underlie covert cognitive processing of incoming information, allowing for a more detailed examination of resources dedicated to stimulus evaluation during sensory and cognitive stages of information processing^107^. In contrast to non-significant RT findings, we observed several significant effects associated with face types while analyzing the neural underpinnings of self-, close-other’s, happy, and neutral faces showed (see below).

On the neural level, we observed that the earliest analyzed ERP component, the N170, was unaffected by the type of faces, as no differences in the analysis of N170 amplitudes were found. Although some studies presented evidence that this component is larger (i.e. more negative) in response to the self-face compared to other faces, familiar or not^35–39^, this pattern of findings has not been consistently confirmed by numerous other studies^9,34,40–49,53,54^.

The N170 reflects an early stage of structural encoding of faces and is not thought to be modulated by face familiarity^27,28,40^. This structural encoding of faces reflects automatic processing, and is relatively immune to external factors (e.g., attention, threat information, and cultural priming) during the early stage of self-face processing^51,108^. Considering the functional role of N170^25,28,109^, our findings which demonstrate a lack of N170 differences between one’s own face and other faces, suggest comparable levels of structural encoding and categorization of faces, along with similar activation of structural face representations for all types of faces (one’s own, close-other’s, happy, neutral), in line with numerous previous studies^9,34,40–50^.

In turn, enhanced N170 in the right hemisphere, observed in the current study, aligns with earlier studies reporting similar lateralization effects for faces^22,110^ and may be linked to functional role of the fusiform gyrus, particularly the right one^111–113^. The fusiform gyrus is not only one of a key structures involved in face perception^114^ but is also a major neural contributor to the N170^115^.

While the early face-selective ERP component (N170) remained unaffected by the type of presented face, such modulation was observed for the N2 and P3 components. The amplitudes of N2 and P3 to the self-face significantly differed from those elicited by close-other’s, happy, and neutral faces. A similar pattern of N2 and P3 findings emerged for the close-other condition as amplitudes of N2 and P3 amplitudes to the close-other’s face significantly differed from those associated with happy and neutral faces. While the amplitudes of N2 to subjectively salient faces (self, close-other’s) were reduced compared to emotional and neutral faces, the amplitudes of P3 were increased. Nonetheless, notable differences between those two types of personally-relevant faces were identified: P3 to one’s own face was higher and N2 was decreased in comparison to a close-other’s face.

Some earlier studies on the topic of self-face processing that investigated the anterior N2 also reported that this ERP component was influenced by the type of presented faces. Specifically, the N2 amplitude was decreased for the self-face when compared to other (both familiar and unfamiliar) faces^39,40,49,53^. The anterior N2 is linked with novelty detection and is increased for novel visual stimuli as compared to familiar ones^116^. In light of this, larger N2 amplitudes to happy and neutral faces than to personally known faces (self, close-other’s) may be related to the fact that those types of faces were novel (unknown) to participants. Moreover, the N2 ERP component is considered an index of the need to exert cognitive control^57^. Therefore, the decreased N2 in the self- and close-other’s face condition may reflect the fact that seeing personally-relevant faces is less surprising than seeing other faces and may indicate a smaller involvement of executive control in the process of visual encoding of those faces^57,59^. The significantly lower N2 to one’s own face than to a close-other’s face may thus indicate that the former demanded even less executive control than the latter.

As far as the late ERPs are concerned, several studies have reported an increase in the P3 amplitude when individuals view their own face in comparison to other faces^9,10,40,49,50,53–55,67,81^. It is noteworthy that such an effect was consistently observed when the control conditions to the self-face included a face of freely chosen^9,10,50,55,56^ or pre-defined (e.g. friend) close person^48,117^. The P3 is related to multiple cognitive functions, including top-down controlled attentional processes^60,118^, as well as cognitive and motivational evaluation^60,68^. Consequently, the increased P3 amplitude to the self-face is typically interpreted as an indicator of increased attentional and motivational processing^67^. The enhanced P3 observed in response to the self-face compared to all other faces (a close-other’s, happy, neutral), as reported in the present study, corroborates findings of previous studies. Additionally, larger P3 amplitudes to the self-face were also found when one’s face was compared with objectively emotional faces^54,75^.

Considering the primary research question, it is essential to highlight that both the self-face and highly familiar and personally-relevant non-self face yielded similar patterns of findings. Specifically, both the self-face and a close-other’s face differed from happy and neutral faces, as revealed by the analysis of N2 and P3 components. Results for both types of personally-relevant and salient faces indicated that their processing differed from emotional faces. Cluster-based permutation tests, employed to contrast the self-face and emotional faces, as well as a close-other’s face and emotional faces, revealed strong and significant differences between the examined conditions. Overall, the outcomes of various methods used to evaluate the processing of personally-relevant faces and emotional faces indicated substantial differences in their neural correlates. Importantly, all of these results consistently demonstrated strong and significant differences between subjectively salient faces and emotional faces over an extended time window: these differences emerged 200 ms after the face images onset and persisted until approximately 1000 ms.

Despite similarities in patterns of ERP findings for the self-face and close-other’s face, the processing of those two types of personally-relevant and subjectively salient faces differed as revealed by results of N2 and P3 analyses and nonparametric cluster-based permutation tests. These results corroborates findings of earlier studies^9,39,50,53,54,56,117^. Specifically, self-face processing was characterized by a reduced N2 over fronto-central sites compared to both friend and stranger faces, although differences between friend and stranger also emerged at this time^39^. Additionally, a decreased N2 and increased P3 was found for the self-face compared to a close-other’s face^53^. One’s own face elicited larger P3 mean amplitudes than friend’s face^117^. This pattern was also evident when comparing the self-face to a freely chosen close-other’s face^9,50,54,56^. Furthermore, the reported results of the nonparametic cluster-based permutation test for self-face vs. a close-other’s face comparison align with findings of other studies that also contrasted these two conditions using this method^10,55^. All these findings collectively indicate that although both those faces are highly familiar, personally-relevant, and salient, the self-face is an exceptional visual stimulus, and its processing differed even from the face processing of subjectively significant person (freely chosen).

An aside to the main topic of the current study, but still intriguing, is the absence of both behavioral and neural differences between happy and neutral faces: amplitudes of N170, N2, and P3 to potentially salient emotional faces did not differ from those to neutral faces. These ERP findings were confirmed by the results of cluster-based permutation test, indicating virtually no differences at any electrode site and any time point. Thus, when confronted with subjectively significant (salient) faces, they were not differentiated, despite eliciting distinct brain responses when processed in different contexts. For instance, the N170 exhibited a robust modulation by emotional facial expression, with emotional faces associated with higher N170 amplitudes than neutral faces^54,89,119^. Similarly, decreased N2 was found also for emotional vs. neutral faces^90,120^, and P3 amplitudes were also influenced by the emotional factor, revealing higher P3 amplitudes to emotional vs. neutral faces^91^. However, an analogous lack of P3 differences between the processing of emotional (happy, fearful) and neutral faces was found in an earlier study that directly compared the processing of the self-face and emotional faces^54^. The lack of neural differentiation of happy vs. neutral faces may be attributed to the notion that emotional feelings, rather than simple emotions, are intrinsically subjective^121,122^. Consequently, viewing the personally-relevant and significant faces may automatically induce the emergence of subjective emotional states, associated with increased brain activity, whereas such subjective emotional states may be absent in the case of objectively emotional faces evaluated by participants as subjectively non-significant stimuli.

Limitations of the current study are outlined below. The set of face images comprised two images of personally-relevant and subjectively significant faces (self, close-other) and two images of unknown faces (happy, neutral). However, an image of a face of a personally known but not self-relevant person (e.g. neighbor) was not included. The inclusion of such a face would broaden the self-relevance scale and allow for the consideration of a gradient of self-relevance and familiarity factors—ranging from the most (self-faces) to the least self-relevant/familiar (unknown faces), with a close-other’s face and a face of personally known but non-significant person in-between. Moreover, all faces featured in the study were of the same sex as the respective participant’s sex. Therefore, it raises the question of whether the reported effects would generalize to faces of other-sex close-others. Additionally, the behavioral task was very simple (detection of faces) and did not required overt discrimination of presented faces. Thus, one may wonder whether reported lack of RTs differences (i.e. specifically—lack of shorter RTs to the self-face in comparison to other faces) would be also found if some recognition or identification tasks were employed. Last but not least, given all RTs in the present study were very short (about 250 ms), participants’ motor response overlapped with the face processing, potentially exerting some influence on the analyzed ERP components.

In conclusion, the processing of subjectively salient faces (one’s own, close-other’s) did not resemble the processing of emotionally positive faces. Notably, self-face prioritization was also observed, as evidenced by substantial differences between one’s own face and all other types of faces (close-other’s, happy, neutral). These findings underscore the critical role of subjective evaluation in determining the saliency factor.

### Supplementary Information

Supplementary Information.

## Data Availability

The data supporting the reported findings are available from the corresponding author upon reasonable request.
