# Supplementary material for: Subjectively salient faces differ from emotional faces: ERP evidence
Source: Sci Rep. 2024 Feb 13;14:3634. doi: 10.1038/s41598-024-54215-5 (PMC10864357; doi:10.1038/s41598-024-54215-5)
Supplement: Supplementary file 1 — Supplementary Information. [file 41598_2024_54215_MOESM1_ESM.pdf]

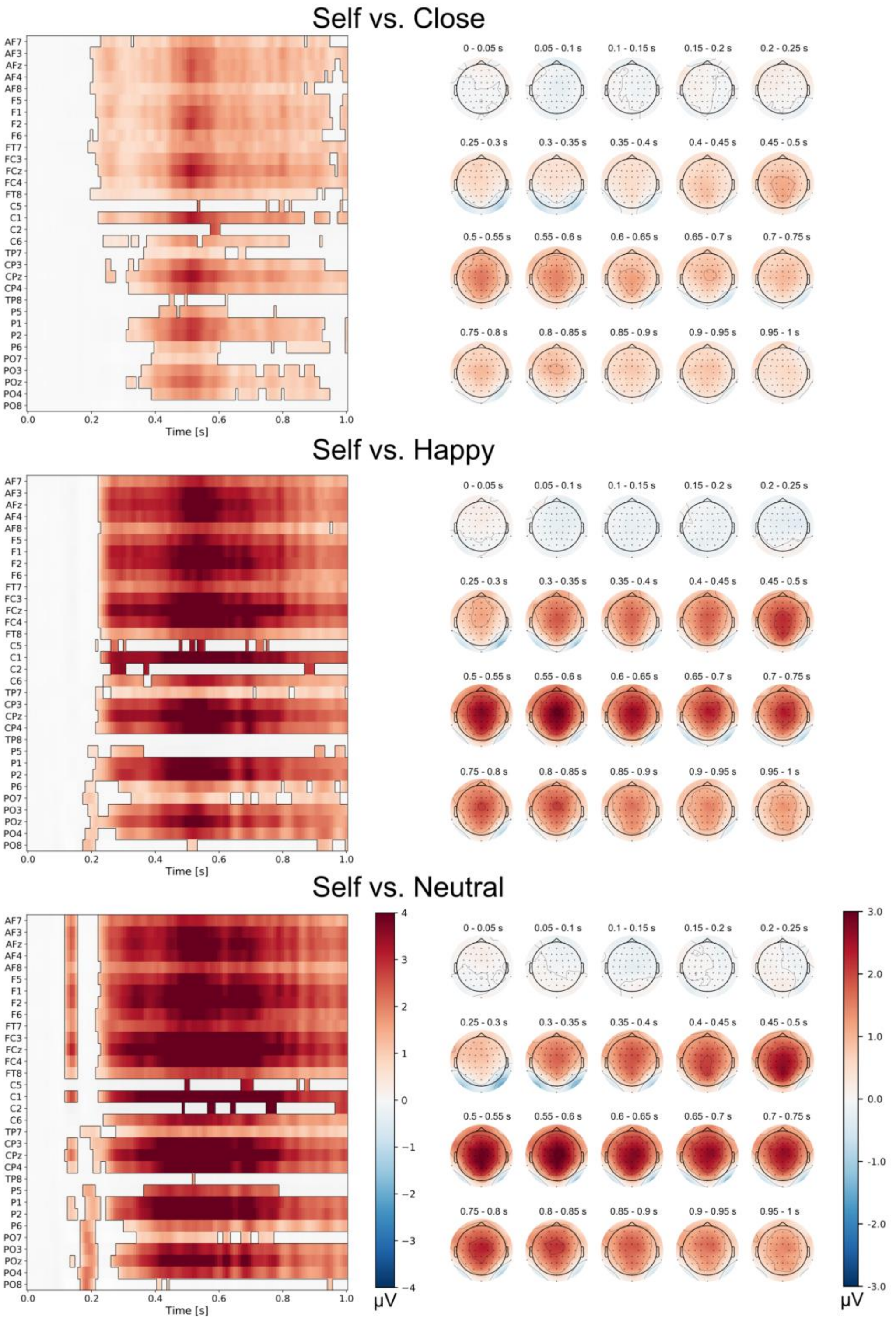

The results of cluster-based permutation tests for the self-face compared to other (close-other’s, happy, neutral) faces (left panel). Statistically significant differences between tested conditions are depicted ($p < 0.05$). The intensity of the color indicated the size of differences between the tested conditions. Results are shown for 32 (from 62) electrode sites (frontal, central, temporal, parietal, occipital, from the top to the bottom, respectively) and all time samples, i.e. from 0 (onset of the face image) till 1 000 ms. Maps depicting the topographical distribution of difference waves are presented for consecutive 50 ms time-windows (right panel).

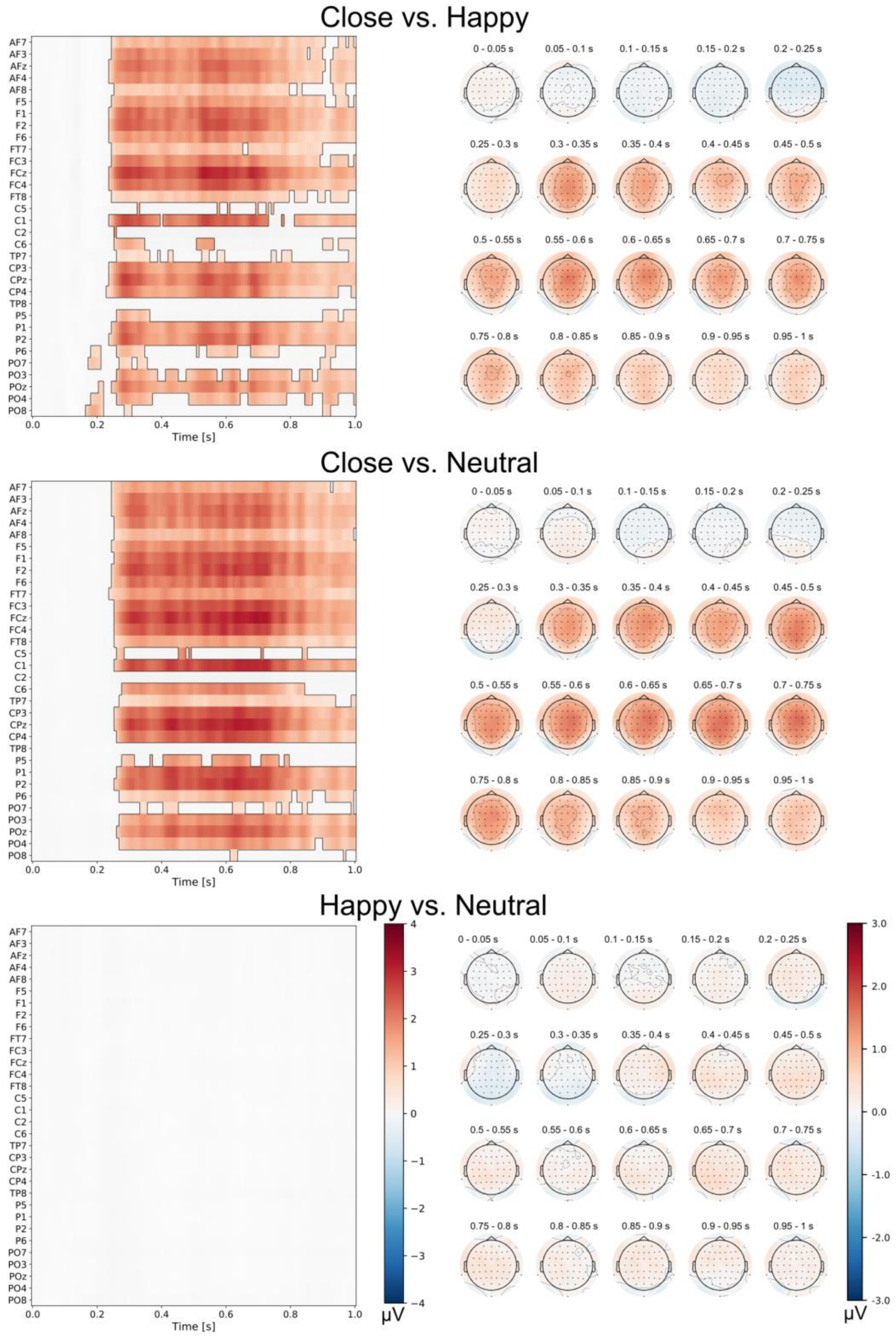

The results of cluster-based permutation tests for a close-other's face compared to other (happy, neutral) faces and for happy vs. neutral face comparison (left panel). Statistically significant differences between tested conditions are depicted ($p < 0.05$). The intensity of the color indicated the size of differences between the tested conditions. Results are shown for 30 (from 62) electrode sites (frontal, central, temporal, parietal, occipital, from the top to the bottom, respectively) and all time samples, i.e. from 0 (onset of the face image) till 1 000 ms (left panel). Maps depicting the topographical distribution of difference waves are presented for consecutive 50 ms time-windows (right panel).
